# Trunk Girdling Increased Stomatal Conductance in Cabernet Sauvignon Grapevines, Reduced Glutamine, and Increased Malvidin-3-Glucoside and Quercetin-3-Glucoside Concentrations in Skins and Pulp at Harvest

**DOI:** 10.3389/fpls.2020.00707

**Published:** 2020-06-09

**Authors:** Giuliano E. Pereira, Emily M. T. Padhi, Raul C. Girardello, Cristina Medina-Plaza, Dave Tseng, Robert C. Bruce, Jesse N. Erdmann, Sahap K. Kurtural, Carolyn M. Slupsky, Anita Oberholster

**Affiliations:** ^1^Brazilian Agricultural Research Corporation-Embrapa Grape & Wine, Bento Goncalves, Brazil; ^2^Department of Viticulture and Enology, University of California, Davis, Davis, CA, United States; ^3^Department of Food Science and Technology, University of California, Davis, Davis, CA, United States; ^4^Department of Nutrition, University of California, Davis, Davis, CA, United States

**Keywords:** amino acids, biosynthesis, grape and wine, ^1^H NMR spectroscopy, metabolome, organic acids, phenolic compounds and sugars, *Vitis vinifera* L.

## Abstract

Girdling is a traditional horticultural practice applied at fruit set or other phenological stages, and is used mostly as a vine management. In grapevines, it is used primarily for table grapes to improve berry weight, sugar content, color, and to promote early harvest. The objective of this study was to evaluate the effect of trunk girdling applied at veraison, in ‘Cabernet Sauvignon’ wine grapes (*Vitis vinifera* L.), on agronomical and physiological parameters during vine development from the onset of ripening (veraison) to harvest, and additionally to quantify the effect of girdling on primary and secondary metabolism. Girdling was applied 146 days after pruning (dap) at veraison, when berry sampling for metabolomics and agronomical evaluations commenced, with a further three sampling dates until harvest, at 156 dap (30% maturation, 10 days after girdling-dag), 181 dap (70% maturation, 35 dag), and 223 dap (commercial harvest, 77 dag). Skin/pulp and seed tissues were extracted separately and metabolomics was performed using one-dimensional proton nuclear magnetic resonance (1D ^1^H NMR) spectroscopy and high performance liquid chromatography (HPLC-DAD). At harvest, girdling significantly increased stomatal conductance (g_s_) in vines, decreased glutamine concentrations, and increased anthocyanin and flavonol concentrations in the skin/pulp tissues of grape berries. Berry weight was reduced by 27% from 181 dap to harvest, and was significantly higher in grapes from girdled vines at 181 dap. Sugars, organic acids, and other amino acids in skin/pulp or seeds were not significantly different, possibly due to extra-fascicular phloem vessels transporting metabolites from leaves to the roots. Using a metabolomics approach, differences between skin/pulp and seeds tissues were meaningful, and a greater number of secondary metabolites in skin/pulp was affected by girdling than in seeds. Girdling is a simple technique that could easily be applied commercially on vine management to improve berry color and other phenolics in ‘Cabernet Sauvignon’ grapes.

## Introduction

‘Cabernet Sauvignon,’ originating from France, is the most important grape variety used for red wines, varietal, or blended in all winegrowing regions worldwide. In Napa Valley, California, the hot and dry Mediterranean climate, defined by mountain ranges and influenced by its proximity to the Pacific Ocean, is propitious for grapes and other fruits to reach a high maturity level (Cayan et al., 2008). In these conditions, grapes at harvest present with high concentrations of sugars, phenolic compounds, high pH, and low acidity. These characteristics require that wineries make adjustments to balance components in order to lower the wine pH to levels suitable for commercial red wines. In most cases, grapes at harvest in Napa Valley are overripe and are sometimes shriveled, resulting in a loss of profitability due to lower berry weights/yield and final volume by concentration, which in turn influences the balance of metabolites in wine (Keller, 2015). Vine management practices should be evaluated for their ability to reduce cycle and harvest time in order to retain grapes with optimal characteristics, which include a high concentration of sugars and phenolics, balanced acidity and pH, and reduced volume loss due to shriveling. Girdling is a simple and easily implemented technique that might improve the quality of grapes intending for winemaking in the Napa Valley region (Williams et al., 2000; Williams and Ayars, 2005).

Girdling is a traditional horticulture practice that involves removing a strip of bark, phloem, and cambium around the trunk or cane of some fruit trees such as mango and vine (Harrell and Williams, 1987; Roper and Williams, 1989; Urban et al., 2004; Ferrara et al., 2014; Gallo et al., 2014; Böttcher et al., 2018). Physiologically, the phloem is responsible for the movement of carbohydrates (sugars and starches) produced by photosynthesizing leaves to developing organs (including the fruit and roots). Phloem sugar is unloaded into the cell vacuole via an apoplastic mechanism requiring the intervention of hexose transporters, and an osmotic gradient translocates phloem to the berries during ripening (Hunter and Ruffner, 2001; Terrier et al., 2005). Removal of a portion of the phloem through girdling prevents the translocation of carbohydrates to the root system, thus supplying more nutrients for fruit growth until the girdle heals (Keller, 2010). The immediate causal effect for plants is to stop the basipetal movement of assimilates through the phloem, which results in an accumulation of carbohydrates above the girdle (Urban et al., 2004). In coniferous trees for wood, girdling is applied at different phenological stages, before, during, or after stem growth, acting as a C sink, and can reveal the dependency of root growth and wood development on current photosynthates throughout the growing season (Rainer-Lethaus and Oberhuber, 2018). Indeed, girdling causes changes in the net rate of CO_2_ assimilation, which is reflected in changes to stomatal conductance (g_s_) and consequently on the behavior of plant performance (Von Caemmerer and Farquhar, 1981; Buckley and Mott, 2013). Stomata exert control over the fluxes of H_2_O vapor and CO_2_ between the leaf and the atmosphere, and adjust their aperture in response to a number of environmental factors, such as girdling, gibberellic acid application, water regimes and seasonal effect on vines. It can be estimated using different parameters, including leaf porometer, thermal imagery and chambers, used at a leaf or whole-plant scale (Roper and Williams, 1989; Leinonen et al., 2006; Buckley and Mott, 2013; Douthe et al., 2018). In grapevines, girdling is normally applied at fruit set or veraison, depending on whether the objective is to increase berry size (at fruit set), reduce cycle duration, or promote metabolite accumulation (at veraison) (Harrell and Williams, 1987; Roper and Williams, 1989; Böttcher et al., 2018). This technique is typically used for table grapes sold as fresh fruit; however, to our knowledge, the influence of girdling on grapevine development, berry weight, and primary and secondary metabolism in ‘Cabernet Sauvignon’ grapes intended for winemaking has not yet been investigated. The application of this technique to winemaking grapevines at veraison should increase the concentration of phenolic compounds and sugars in grape berries, and reduce the growing season to allow for early harvest, thus avoiding volume loss during the winemaking process (Keller et al., 2006).

In wine grapes, metabolites are found mainly in the skin, pulp (or flesh), and seeds of the berries, including sugars, organic acids, amino acids, and some polyphenols such as flavonols and hydroxycinnamic acids (Ollat et al., 2002; Ribéreau-Gayon et al., 2006; Carbonneau et al., 2015). In non-teinturier grape varieties, anthocyanins are only found in the skins, as well as flavanols (also called flavan-3-ols) and flavonols, while the largest portion of the flavanols are located in the seeds (Kennedy et al., 2000; Cerpa-Calderón and Kennedy, 2008; Ali et al., 2010). Flavan-3-ols are present as monomers and various oligomers called proanthocyanidins, collectively called grape tannins. The accumulation of the primary metabolite sugars and organic acids is well known in the literature; however, less is known regarding the development of secondary metabolites in different grape tissues, as these processes are regulated by different genes and pathways (Coombe and McCarthy, 2000; Hunter and Ruffner, 2001; Ollat et al., 2002; Keller, 2010; Cohen et al., 2012; Rienth et al., 2014). Previous studies have shown that tannin biosynthesis occurs mostly during the early stages of berry development, while the ripening phase is characterized by polymerization reactions and other alterations to existing tannin units (Downey et al., 2006; Keller, 2010). Furthermore, study of the grape ripening process is difficult, due to the heterogeneity of berries in the grape bunches (Kuhn et al., 2014; Reshef et al., 2019). Viticulturists and enologists can optimize the composition of sugars, organic acids, and amino acids in wine grapes by adjusting management practices during vine development or during winemaking (Ribéreau-Gayon et al., 2006; Carbonneau et al., 2015).

Metabolomics describes the metabolic composition of samples present at diverse concentrations, while the term metabolome is the multivariate sum of these components (Fiehn, 2002; Zhang et al., 2011; Nicholson et al., 2012; Xia et al., 2015). These analyses are considered quantitative measurements of the dynamic multiparametric metabolic response of living systems to environmental stimuli or genetic modification (Fiehn et al., 2000; Roessner et al., 2001; Holmes et al., 2019). Metabolic phenotyping involves the comprehensive analysis of biological fluids or tissue samples (Roullier-Gall et al., 2014).

Different analytical methods have been used to study the influence of natural or induced factors on model plants, vine development, and metabolic compounds in grapes/wines by gas chromatography mass spectroscopy (GC-MS) (Fiehn et al., 2000), proton nuclear magnetic resonance (^1^H NMR) spectroscopy (Krishnan et al., 2005; Pereira et al., 2006a, b; Lima et al., 2010; Zhang et al., 2012; Gallo et al., 2014; Peterson and Waterhouse, 2016; Cassino et al., 2019), and high performance liquid chromatography (HPLC) (Peng et al., 2002; Oberholster et al., 2013; Hernández-Hierro et al., 2014; Garrido-Bañuelos et al., 2019a). ^1^H NMR spectroscopy is a powerful tool that allow for the simultaneous determination of metabolites from different groups of organic compounds, such as sugars, organic acids, amino acids, some polyphenols, and vitamins in a single run, and uses a targeted or non-targeted approach to describe metabolic profiles from different conditions and experiments (Pereira et al., 2006a; Fotakis et al., 2013; Godelmann et al., 2013; Lloyd et al., 2015; Pinu, 2018). ^1^H NMR spectroscopy is also used to quantify metabolites in cell or tissue extracts without the necessity of *a priori* knowledge of the sample composition (Gallo et al., 2014). However, the determination of most phenolic compounds is difficult using one dimension ^1^H NMR spectroscopy, due to their molecular complexity (Pereira et al., 2006b). Instead, HPLC is largely used to determine phenolics in grapes and wines, with easy identification of the anthocyanins, flavonols, flavanols and hydroxycinnamic acids in a single run at different wavelengths (Peng et al., 2002; Oberholster et al., 2013; Garrido-Bañuelos et al., 2019b; Girardello et al., 2019).

The purpose of this study was to evaluate the effect of trunk girdling, applied at veraison, on agronomical and physiological parameters during vine development, and to determine how primary and secondary metabolites in the skin/pulp and seed tissues of ‘Cabernet Sauvignon’ grapes are altered over four different phenological stages, using a metabolomics approach. It is expected that trunk girdling will increase the content of sugars and phenolic compounds at harvest, which may enable an earlier harvest date in the future, due to potential earlier ripening, thereby avoiding volume losses and must/juice corrections by wineries associated with the long growing season in Napa Valley.

## Materials and Methods

### Field Procedure and Agronomical Parameters

This study was carried out during the 2018 growing season at the University of California Experimental Station in Oakville, Napa County, CA, United States (38°25′ N; 122°24′ W). The vineyard was planted in 2012 with *V. vinifera* L. ‘Cabernet Sauvignon’ FPS 08 (Foundation Plant Services, UC Davis), grafted onto rootstock 110R. Plant spacing was 2.0 × 2.4 m (vine × row) in Northeast-Southwest oriented rows. Grapevines were trained to bilateral cordons and vertical-shoot-positioned trellis, and the vineyard was drip-irrigated with two pressure compensating emitters per plant delivering 2.0 L h^–1^ each.

**FIGURE 1:**
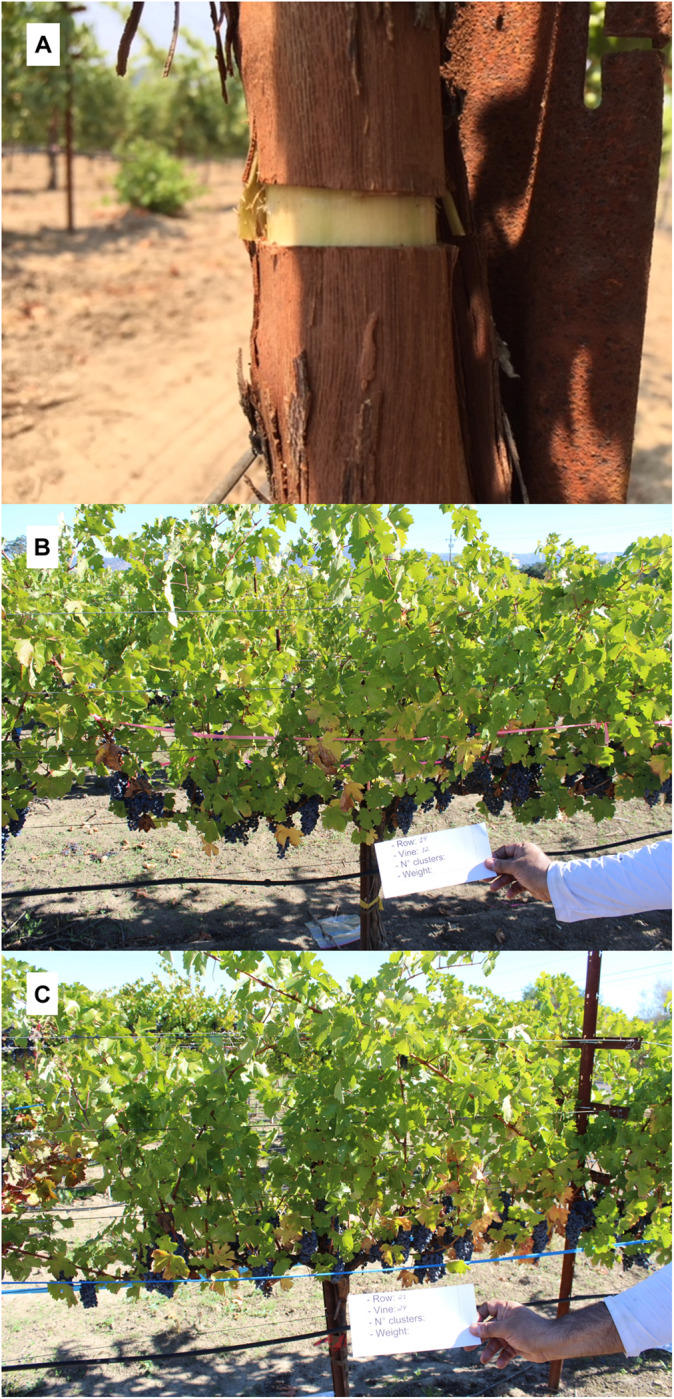
Experimental procedures conducted at UCDavis. **(A)** example of a 1 cm thick girdle made on *Vitis vinifera* L. vines 146 days after pruning (dap). **(B)** a non-girdled vine at harvest. **(C)** a girdled vine at harvest.

The vineyard was composed of 12 rows containing 25 vines per row, and 8 vines were selected randomly per treatment (8 biological replicates for girdled and 8 for non-girdled vines), in different parts of the plot, to account for soil variability. Vines were pruned on March 7th 2018 and evaluations began at veraison, on July 31st 2018, 146 days after pruning (dap), when the first girdle was applied (Figure 1A). An entire ring of bark was removed, approximately 1 cm of thickness, all around the trunk, 10 cm below bilateral arms formation of the vines. Berry samples were collected (40 berries/vine, and 320 berries per treatment) for analyses, kept on ice in a cooler, then put in the liquid nitrogen prior storage in a freezer at −80°C, as described in sample preparation. The second sampling of 40 berries was carried out at 30% of berry maturation, on August 10th, 156 dap, 10 days after girdling (dag), as described previously. The second girdling was applied on August 31st, thirty days after first girdling, in order to ensure the girdling technique was initiated correctly and to avoid photo-assimilated transportation to the roots (Rainer-Lethaus and Oberhuber, 2018). The third sampling took place on September 4th, 181 dap (35 dag) at 70% of berry maturation, and the fourth and last sampling took place at harvest, on October 16th, 223 dap (77 dag) (Figures 1B,C). Stomatal conductance (g_s_) was measured at all four phenological stages described before, with a leaf porometer (METER Group, Inc., Pullman, WA, United States), and was evaluated in two different leaves per vine, or 16 leaves per treatment. Two fully expanded sun exposed leaves from the top of the canopy were measured as previously described (Roper and Williams, 1989; Williams and Ayars, 2005).

### Chemicals and Standards

Ethanol (96%), methanol (reagent grade), acetonitrile (HPLC grade), (+)-catechin hydrate (98%), (−) epicatechin (90%), p-coumaric acid (98%), ferulic acid (99%), caffeic acid (98%), quercetin (95%), gallic acid monohydrate (99%), syringic acid (98%), and vanillic acid (97%) were purchased from Sigma Aldrich (St. Louis, MO, United States). Malvidin-3-O-glucoside (95%) was purchased from Extrasynthese (Genay, France). Phosphoric acid (88%) (HPLC grade) was purchased from Fisher Scientific (Pittsburgh, PA, United States). Deionized water was prepared in-house to a final purity of 18.2 MΩ⋅cm. D_2_O (99.9%) was purchased from Cambridge Isotope Laboratories, Inc. (Tewksbury, MA, United States). 3-(trimethylsilyl)-1-propanesulfonic acid-d6 (DSS-d6) was purchased from Chenomx (Edmonton, Alberta, Canada). NMR tubes were purchased from Bruker BioSpin (Billerica, MA, United States). Internal standard 2,2,3,3,4,4-d6-3-(trimethylsilyl)-1-propane sulfonic acid (DSS-d6) and sodiumazide (NaN3) in D_2_O was from Chenomx Inc. (Edmonton, Alberta, Canada). Sodium hydroxide and hydrochloric acid solutions were purchased from Fisher Scientific (Fair Lawn, NJ, United States).

### Sample Preparation

From the 40 ‘Cabernet Sauvignon’ berries initially collected per vine per phenological stage of each treatment, 20 berries were used to determine pH, total soluble solids, total acidity, and berry weight on the same day as collection. The remaining berries were put in the liquid nitrogen then stored at −80°C until further analysis.

### Berry Tissue Extraction

Of the remaining 20 berries stored at −80°C, 10 berries were used for metabolomics analyses (^1^H NMR and HPLC). The remaining 10 berries were kept at −80°C for long-term storage, and eventually were discarded. First, ten berries were weighed and then split in half with a scalpel to separate seeds from the skin/pulp. Both tissues (skin/pulp and seeds) were weighed, seeds were counted, and both tissues were ground separately with ethanol for 3 min using a T18 digital ULTRA-TURRAX^®^ (IKA^®^ Works, Inc., Wilmington, NC, United States). Each sample was placed inside a cooler and mixed (1 h, 10°C) with a magnetic multiple stirrer with external control unit (2mag Magnetic Motion, Muenchen, Germany) (Pereira et al., 2006a, b). Then, samples were centrifuged at 4000 rpm (1792 relative centrifugal force-rcf) for 10 min and stored at −80°C until metabolomics analyses by ^1^H NMR spectroscopy and HPLC-DAD.

### 1D ^1^H NMR Spectroscopy

Aliquots (1 mL) of ethanolic extracts containing ground skins/pulp or seeds were dried under vacuum for 24 h at room temperature (20°C ± 2). Then, samples were suspended with 1 mL of D_2_O and dried again under vaccuum for 24 h to remove/reduce ethanol and water signals. Samples were dissolved in 1 mL of 10 mM potassium phosphate buffer (pH 6.8 ± 0.1) and centrifuged (5 min, 4°C, 14 krcf) using an Eppendorf^®^ Model 5415R microcentrifuge (Eppendorf North America, Hauppauge, NY, United States). A portion of the supernatant (585 μL) was combined with 65 μL of internal standard containing 5 mM 3-(trimethylsilyl)-1-propanesulfonic acid-d6 (DSS-d_6_), NaN_3_, and D_2_O. The final concentrations were 0.5 mM DSS-d6, 0.02% NaN3, and ∼10% D_2_O. The pH of the sample was adjusted to 6.8 ± 0.1 with 1 N NaOH or HCl and 600 μL of the subsequent mixture was transferred to 5 mm NMR tubes and stored at 4°C until ^1^H NMR data were acquired (within 24 h of sample preparation) (Chin et al., 2014). The 1D ^1^H NMR spectra of the aqueous samples of skins/pulp and seeds were acquired at 298 K using the Bruker “noesypr1d” experiment on a Bruker Avance 600 MHz NMR spectrometer equipped with a SampleJet. The acquisition parameters were: 12 ppm sweep width, 2.5 s acquisition time, 2.5 s relaxation delay, and 100 ms mixing time. Water saturation was applied during the relaxation delay and mixing time. The resulting spectra were zero-filled to 128,000 data points and an exponential apodization function corresponding to a line-broadening of 0.5 Hz was applied. Spectra were processed for metabolite identification and quantification using the Chenomx Inc. NMR Suite Processor version 8.2 (Edmonton, AB, Canada). Each spectrum was acquired in approximately 12 min.

### HPLC-DAD

Ethanolic extracts (1 mL) of skins/pulp and seeds were centrifuged (5 min, 4°C, 10 krcf), and the resulting supernatant was transferred to HPLC vials for analysis by HPLC-DAD (Peng et al., 2002; Oberholster et al., 2013). For the skins/pulp, four wavelengths were used in the same run, at 280 nm, to determine flavanols, 320 nm for hydroxycinnamic acids, 360 nm for flavonols and 520 nm for anthocyanins. Seeds were analyzed at 280 nm to identify and quantify flavanols (Girardello et al., 2019). Samples were analyzed by RP-HPLC using an Agilent 1260 Infinity equipped with a PLRP-S 100A 3 μM 150 × 4.6 mm column (Agilent Technologies, Santa Clara, CA, United States) at 35°C, an auto sampler with temperature control at 8°C and diode array detector, according to previous studies (Peng et al., 2002; Oberholster et al., 2013). Each chromatogram was acquired in approximately 105 min, and peaks were identified and qualified using ChemStation software (B.04.03, 2011).

### Statistical Analyses

All results acquired from agronomical, physiological, physicochemical, NMR, and HPLC data were evaluated for normality using histograms and the Shapiro−Wilk test. Differences between girdling (G+) and non-girdled (G-) groups were evaluated using the Mann-Whitney *U*-test and results were considered significant if *p* < 0.05. Principal components analysis (PCA) was performed with mean centering and unit variance scaling. The quality of the models was judged by the goodness-of-fit parameter (R^2^X or R^2^Y). For ^ 1^H NMR data, the chemical shifts and metabolite identifications were assigned with literature and the Chenomx Inc. database (Chin et al., 2014; Kortesniemi et al., 2016). All figures and statistical procedures were carried out in R Version 3.5.1 (R Core Team, 2012).

## Results

### Agronomical Data

Agronomical parameters evaluated in vines, berries, clusters, and shoots at different phenological stages are shown in Table 1. Significant differences were observed for stomatal conductance of the vines (g_s_) at 181 dap (35 dag, or 70% maturation) and 223 dap (77 dag, or harvest date), and pH at harvest (223 dap). At 70% maturation g_s_ was higher in G(−), while at harvest, G(+) presented the highest values (190 mmol H_2_O m^–2^ s^–1^, vs. 128 mmol H_2_O m^–2^ s^–1^ for G(−). The pH of grapes at harvest from G(+) was slightly but significantly lower than pH of grapes from G(−). No differences were found for°Brix, total acidity, berry weight, number of berries per cluster, weight of berry clusters, number and weight of shoots, and Ravaz Index at harvest.

**TABLE 1:** Agronomical parameters measured in ‘Cabernet Sauvignon’ vines, grapes, clusters, and shoots at different phenological stages^1^, from two groups^2^.

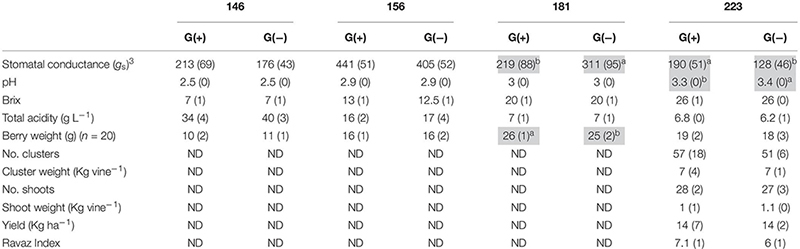

*^1^Phenological stages represented as days after pruning (DAP), where 146, veraison; 156, 30% maturation; 181, 70% maturation; 223, harvest; ^2^Grapevines received girdling application, G(+), or were non-girdled control, G(−); ^3^Stomatal conductance measured in mmol H_2_0 m^–2^s^–1^. ND, not determined. Measures of cluster weight, number and shoot weight, yield, and Ravaz index were collected at harvest (223 DAP) only. Values are median (interquartile range, IQR). Differences between groups at each phenological stage were evaluated using the Mann Whitney *U*-test. Shaded groups with different letters are statistically significant (*p* < 0.05).*

### Primary and Secondary Metabolites Determined Through 1D ^1^H NMR Spectroscopy

Twenty metabolites were identified and quantified in skins/pulp of Cabernet Sauvignon grapes, including sugars, organic acids, carboxylic acids, amino acids, phenolics, and one vitamin (Table 2). Except for carboxylic acids, small but significant differences were observed in all classes of primary and secondary metabolites, at different phenological stages, including fructose and glucose at 30% maturation (156 dap, and 10 dag), tartaric acid (156 dap), malic acid (181 dap, 35 dag), glutamine (156 dap and 10 dag, and 223 dap and 77 dag), threonine (146 and 181 dap), epicatechin (223 dap). Twenty-one metabolites were identified and quantified in seed extracts, including sugars, organic acids, carboxylic acids, amino acids, phenolics, and one vitamin (Table 3). Compared to skin/pulp, fewer significant results were observed in seeds. There were no differences in sugars, except for glucose at 181 dap, which was reduced in G(+) grapevines. Other small differences in tartaric acid, pyruvic acid, succinic acids, and alanine were also observed.

**TABLE 2:** Metabolite profiling determined by 1D ^1^H NMR spectroscopy in skin/pulp of ‘Cabernet Sauvignon’ grapes^1^ at different phenological stages^2^, from two treatments^3^.

	**146**		**156**		**181**		**223**	
	**G(+) *n = 8***	**G(−) *n = 8***	**G(+) *n = 8***	**G(−) *n = 8***	**G(+) *n = 7^**‡**^***	**G(−) *n = 8***	**G(+) *n = 8***	**G(−) *n = 8***
Fructose	7343 (3242)	6439 (4375)	34317 (4450)^b^	37293 (1352)^a^	61695 (1541)	62269 (2823)	85309 (5086)	85900 (4031)
Glucose	12879 (2601)	11612 (5275)	39787 (4783)^b^	42550 (771)^a^	65842 (878)	66106 (2564)	87102 (6507)	89318 (4682)
Sucrose	96 (83)	107 (48)	1151 (276)	1315 (246)	2569 (1095)	2418 (1351)	4746 (1398)	4280 (915)
Total sugars	20318	18158	75255	81158	130106	130793	177157	179498
Malic	12127 (1438)	12982 (977)	4966 (1188)	4767 (609)	824 (68)^b^	1042 (189)^a^	353 (142)	417 (92)
Tartaric	4898 (699)	4807 (721)	2507 (246)^a^	1995 (481)^b^	539 (144)	500 (113)	376 (169)	421 (217)
Total organic acids	17025	17789	7473	6762	1363	1542	729	838
Formic	1 (0)	1 (0)	1 (0)	1 (0)	2 (0)	2 (0)	2 (0)	2 (0)
Succinic	13 (5)	12 (2)	7 (1)	7 (1)	1 (0)	2 (1)	1 (0)	1 (1)
Total carboxylic acids	14	13	8	8	3	4	3	3
Alanine	11 (4)	11 (2)	11 (3)	10 (2)	14 (2)	12 (1)	6 (2)	7 (3)
Arginine	7 6)	7 (3)	10 (5)	9 (4)	10 (5)	8 (3)	6 (6)	8 (3)
GABA	26 (8)	26 (14)	32 (9)	37 (7)	39 (5)	38 (7)	39 (8)	45 (12)
Glutamine	30 (8)	24 (2)	24 (8)^b^	30 (7)^a^	13 (4)	11 (4)	4 (2)^b^	7 (1)^a^
Isoleucine	1 (0)	1 (0)	2 (1)	2 (1)	4 (1)	2 (1)	12 (1)	13 (2)
Leucine	1 (0)	1 (0)	2 (1)	2 (1)	3 (2)	2 (1)	11 (1)	12 (3)
Proline	4 (4)	6 (3)	29 (24)	42 (15)	349 (89)^a^	309 (79)^b^	841 (152)	927 (117)
Threonine	22 (7)^a^	12 (5)^b^	15 (3)	15 (3)	19 (4)^a^	12 (1)^b^	14 (4)	13 (2)
Tyrosine	2 (1)	2 (1)	3 (1)	3 (1)	4 (1)	4 (1)	17 (6)	15 (4)
Valine	1 (1)	2 (0)	3 (1)	3 (1)	7 (2)	5 (1)	21 (3)	21 (3)
Total amino acids	105	92	131	153	462	403	971	1068
Epicatechin	2 (1)	2 (1)	3 (2)	4 (1)	−	−	3 (0)^a^	2 (1)^b^
Gallic acid	2 (1)	2 (0)	1 (0)	1 (0)	−	−	1 (0)	1 (1)
Total phenolics	4	4	4	5	−	−	4	3
Choline	5 (2)	5 (4)	14 (2)	14 (1)	15 (1)	15 (2)	18 (2)	18 (1)

*^1^Values are median (interquartile range, IQR) mg Kg^–1^ of skin/pulp fresh weight; ^2^Phenological stages represented as days after pruning (DAP), where 146, veraison; 156, 30% maturation; 181, 70% maturation; 223, harvest; ^3^Grapevine received girdling application, G(+), or were non-girdled control, G(−). Differences between groups at each phenological stage were evaluated using the Mann Whitney *U*-test. Shaded groups with different letters are statistically significant (*p* < 0.05). *^‡^*One sample was lost during processing.*

**TABLE 3:** Metabolite profiling determined by 1D ^1^H NMR spectroscopy in seeds of ‘Cabernet Sauvignon’ grapes^1^ at different phenological stages^2^, from two treatments^3^.

	**146**		**156**		**181**		**223**	
	**G(+) *n = 8***	**G(−) *n = 8***	**G(+) *n = 8***	**G(−) *n = 8***	**G(+) *n = 7^**‡**^***	**G(−) *n = 8***	**G(+) *n = 8***	**G(−) *n = 8***
Fructose	2132 (592)	2167 (441)	7000 (1370)	6843 (1194)	10030 (382)	10936 (855)	25063 (3301)	23615 (3042)
Glucose	1913 (554)	1916 (619)	5934 (534)	6027 (1384)	9510 (883)^b^	11311 (809)^a^	25529 (2356)	24576 (3985)
Sucrose	5001 (1146)	5175 (1349)	8852 (2492)	9524 (1209)	15845 (2055)	16084 (2794)	12774 (2351)	12626 (891)
Total sugars	9046	9258	21786	22394	35385	38331	63366	60817
Malic	113 (19)	107 (10)	86 (21)	82 (31)	48 (7)	44 (15)	40 (14)	33 (7)
Tartaric	132 (52)	162 (43)	128 (45)	135 (38)	130 (11)^b^	163 (50)^a^	280 (80)	199 (44)
Total organic acids	245	269	214	217	178	207	320	232
Formic	−	−	5 (5)	3 (1)	114 (26)	132 (14)	179 (59)	196 (15)
Pyruvic	3 (1)^b^	5 (1)^a^	4 (1)	5 (1)	4 (2)	4 (3)	5 (2)	5 (2)
Succinic	18 (4)^a^	14 (5)^b^	44 (14)	44 (4)	42 (5)	41 (9)	13 (5)	15 (4)
Total carboxylic acids	21	19	53	52	160	177	197	216
Alanine	146 (25)^a^	115 (22)^b^	175 (34)	198 (33)	114 (25)	115 (37)	50 (29)	44 (14)
Arginine	11 (5)	12 (3)	13 (6)	13 (6)	9 (2)	10 (2)	14 (4)	15 (3)
GABA	184 (18)	154 (59)	166 (33)	169 (37)	140 (33)	160 (38)	123 (90)	109 (72)
Glutamine	283 (86)	348 (96)	96 (21)	127 (44)	51 (11)	65 (29)	36 (14)	38 (5)
Isoleucine	−	−	3 (2)	3 (1)	1 (1)	2 (1)	2 (1)	3 (1)
Leucine	52 (11)	53 (5)	47 (10)	44 (9)	20 (5)	22 (8)	20 (5)	15 (4)
Phenylalanine	42 (12)	44 (9)	31 (9)	30 (9)	35 (2)	38 (3)	31 (5)	28 (5)
Proline	37 (13)	48 (9)	70 (11)	78 (25)	129 (29)	134 (36)	306 (57)	271 (36)
Threonine	26 (11)	26 (4)	27 (7)	27 (6)	24 (3)	22 (5)	16 (3)	13 (5)
Tyrosine	128 (20)	128 (15)	110 (16)	100 (18)	30 (5)	35 (5)	28 (14)	19 (4)
Valine	45 (12)	45 (4)	51 (12)	47 (9)	46 (8)	42 (11)	41 (7)	37 (9)
Total amino acids	954	973	789	736	599	645	667	592
Epicatechin	1665 (254)	1615 (232)	1377 (352)	1595 (545)	581 (206)	567 (150)	284 (41)	271 (103)
Choline	4 (1)	3 (1)	15 (5)	14 (2)	23 (4)	27 (9)	23 (6)	24 (5)

*^1^Values are median (interquartile range, IQR) mg Kg^–1^ of seeds fresh weight; ^2^Phenological stages represented as days after pruning (DAP), where 146, veraison; 156, 30% maturation; 181, 70% maturation; 223, harvest; ^3^Grapevine received girdling application, G(+), or were non-girdled control, G(−). Differences between groups at each phenological stage were evaluated using the Mann Whitney *U*-test. Shaded groups with different letters are statistically significant (*p* < 0.05). *^‡^*One sample was lost during processing.*

### Secondary Metabolites Determined by HPLC-DAD

Twenty-three phenolic metabolites were identified and quantified in skin/pulp, with two unknown compounds (Table 4). Significant differences were observed in all classes of phenolics, but at different phenological stages. Concentrations of the flavanol epicatechin gallate were higher in G(−) vines at veraison, and lower at 156 dap, but not at harvest. The flavonols quercetin-3-glucose, quercetin-3-glucuronide and quercetin-3-galactoside were higher in G(+) vines at 156 dap, while quercetin-3-glucose (26.3 vs. 19.2 mg/kg fresh weight) and an unknown flavonol-1 (7.3 vs. 5.8 mg/kg fresh weight) were higher in G(+) at harvest. The response of the anthocyanins was varied, as some metabolites were higher in non-girdled vines, while others in girdled vines, at different phenological stages. The compounds cyanidin-3-glucoside, delphinidin-3-glucoside, peonidin-3-glucoside, petunidin-3-glucoside, and the acetyl acetylated and p-coumaroyl acylated forms of delphinidin-3-glucoside, were significantly higher in non-girdled vines at 70% maturation, but no differences were found at harvest. The most important anthocyanin in *Vitis vinifera* L. is malvidin-3-glucoside, and girdling appeared to increase the concentration of this compound at harvest (181.7 vs. 167.1 mg/kg fresh weight). A similar finding was observed in G(+) vs G(−) vines for malvidin-3-acetylglucoside (73.1 vs. 63.4 mg/kg fresh weight), and p-coumaroyl acylated forms (36.5 vs. 17.7 mg/kg fresh weight). Berries grown on girdled grapevines also presented with higher concentrations at different phenological stages and at harvest of peonidin-p-coumaroyl acetylated (4.8 vs. 4.2 mg/kg fresh weight), hydroxycinamic acid caftaric (6.1 vs. 4.0 mg/kg fresh weight), as well as polymeric phenol (1092.6 vs. 671.8 mg/kg fresh weight) (Table 4). Six phenolic compounds were identified and quantified in seeds, none of which were significant, except for compound procyanidin “B1” at 146 dap (Table 5).

**TABLE 4:** Phenolic compounds determined by HPLC-DAD in skin/pulp of ‘Cabernet Sauvignon’ grapes^1^ at different phenological stages^2^, from two treatments^3^.

	**146**		**156**		**181**		**223**	
	**G(+) *n = 8***	**G(−) *n = 8***	**G(+)*n = 8***	**G(−) *n = 8***	**G(+)*n = 7^**‡**^***	**G(−) *n = 8***	**G(+)*n = 8***	**G(−) *n = 8***
Catechin	17.7 (6.5)	19.2 (3.9)	2.5 (1.5)	2.6 (1.2)	4.1 (0.7)	4.7 (0.4)	6.9 (0.7)	8.0 (1.4)
Epicatechin	3.0 (0.7)	2.7 (0.7)	2.0 (0.3)	1.9 (0.4)	7.5 (2.2)	6.7 (2.4)	26.3 (2.6)	26.3 (4.9)
Epicat. gallate	6.2 (1.7)^b^	7.8 (1.3)^a^	2.8 (1.6)^a^	1.3 (0.6)^b^	2.1 (0.7)	1.5 (0.6)	3.1 (1.2)	2.5 (0.3)
Epigallocatechin	4.9 (1.7)	5.0 (1.3)	1.6 (0.4)	1.3 (0.3)	2.3 (0.8)	2.1 (2.2)	4.0 (2.0)	4.7 (2.5)
Total Flavanols	31.8	34.7	8.9	7.4	15.9	15.1	40.4	41.6
Querc-3-gluc.	6.5 (4.7)	5.4 (3.4)	7.9 (2.8)^a^	3.9 (2.9)^b^	13.9 (5.0)	11.5 (3.4)	26.3 (6.1)^a^	19.2 (3.0)^b^
Quer-glucur.	33.2 (2.7)	30.67 (7.4)	25.4 (7.9)^a^	13.8 (8.6)^b^	11.7 (3.8)	9.9 (2.9)	17.3 (4.7)	14.4 (1.9)
Querc-galact.	1.8 (1.1)	1.6 (0.7)	2.9 (0.5)^a^	1.9 (0.9)^b^	4.4 (1.1)	4.8 (0.8)	7.4 (1.8)	6.5 (1.0)
Quer-rhamn.	0.3 (0.6)	0.4 (0.2)	0.8 (0.4)	0.6 (0.5)	3.7 (1.1)	3.4 (0.9)	8.7 (2.2)	7.1 (0.9)
Unknown 1	0.6 (0.2)	0.5 (0.1)	0.9 (0.3)	1.0 (0.3)	3.6 (0.8)	3.7 (0.4)	7.3 (1.0)^a^	5.8 (1.9)^b^
Unknown 2	ND	ND	3.9 (1.8)	3.3 (1.8)	14.4 (1.7)	13.0 (2.8)	26.1 (3.7)	24.9 (4.9)
Total Flavonols	42.5	38.5	39.5	24.7	51.7	446.4	93.3	78.16
Cya-3-gluc.	−	−	2.1 (2.1)	2.6 (1.7)	1.9 (0.4)^b^	3.4 (0.8)^a^	3.1 (1.3)	4.2 (0.9)
Delph-3-gluc.	−	-	7.3 (5.2)	8.4 (3.3)	21.3 (3.7)^b^	27.1 (1.5)^a^	32.9 (5.1)	38.8 (9.8)
Malv-3-gluc.	−	−	23.2 (3.7)	24.3 (6.6)	78.4 (10.4)	84.9 (8.1)	181.7 (8.1)^a^	167.1 (22.1)^b^
Peo-3-gluc.	−	−	6.6 (5.2)	6.9 (3.3)	11.6 (1.6)^b^	14.3 (0.7)^a^	21.5 (2.8)	23.8 (4.7)
Pet-3-gluc.	−	−	5.4 (3.2)	5.9 (2.0)	13.85 (2.4)^b^	16.4 (0.7)^a^	23.6 (2.0)	26.7 (6.7)
Delph-3-acet.	−	−	2.8 (2.2)	4.1 (1.2)	6.8 (1.8)^b^	9.8 (1.0)^a^	11.0 (2.3)	12.7 (3.3)
Malv-3-acet.	−	−	13.6 (1.8)	16.0 (2.9)	36.7 (8.1)	38.7 (4.1)	73.1 (2.9)^a^	63.4 (4.2)^b^
Peo-3-acet.	−	−	3.3 (1.6)	3.6 (1.1)	4.1 (0.8)	5.5 (0.6)	6.7 (1.0)	7.3 (1.1)
Pet-3-acet.	−	−	2.6 (1.6)	3.4 (0.9)	5.8 (1.3)	7.7 (0.6)	9.7 (0.9)	11.1 (2.3)
Delph-3-pcoum.	−	−	1.0 (0.5)	1.1 (0.3)	2.3 (0.4)^a^	2.8 (0.2)^a^	3.7 (0.3)	3.8 (0.8)
Malv-3-pcoum.	−	−	5.2 (0.7)	4.9 (0.8)	14.6 (2.1)	13.8 (1.1)	36.5 (3.3)^a^	17.7 (10.8)^b^
Peo-3-pcoum.	−	−	2.8 (1.0)	2.5 (0.8)	3.6 (0.8)	3.9 (0.5)	4.8 (1.3)^a^	4.2 (0.6)^b^
Pet-3-pcoum.	−	−	0.3 (0.1)	0.3 (0.1)	0.6 (0.1)	0.6 (0.3)	1.6 (0.2)	1.5 (0.2)
Total Anthocyanins	−	−	76.5	84.3	201.7	221.2	410.2	382.4
Caftaric acid	47.5 (27.1)	44.8 (10.8)	14.8 (3.5)^a^	11.9 (2.8)^b^	3.1 (1.0)	2.9 (1.7)	6.1 (0.6)^a^	4.0 (1.4)^b^
Polymeric phenols	1350.99 (342.1)^a^	1052.2 (275.2)^b^	866.3 (113.8)	533.2 (106.5)	600.9 (144.3)^a^	370.1 (42.7)^b^	1092.6 (178.1)^a^	671.8 (222.4)^b^
Polymeric pigments	1.9 (0.9)	1.6 (0.6)	0.7 (0.1)	0.9 (0.3)	1.4 (0.3)	1.3 (0.1)	4.7 (0.6)	4.1 (0.9)
∑ all phenolics	1550	1246	1131	781	1152	947	2206	1697

*^1^Values are median (interquartile range, IQR) mg Kg^–1^ of skin/pulp fresh weight; ^2^Phenological stages represented as days after pruning (DAP), where 146, veraison; 156, 30% maturation; 181, 70% maturation; 223, harvest; ^3^Grapevine received girdling application, G(+), or were non-girdled control, G(−). Differences between groups at each phenological stage were evaluated using the Mann Whitney *U*-test. Shaded groups with different letters are statistically significant (*p* < 0.05). *^‡^*One sample was lost during processing.*

**TABLE 5:** Phenolic compounds determined by HPLC-DAD in seeds of ‘Cabernet Sauvignon’ grapes^1^ at different phenological stages^2^, from two treatments^3^.

	**146**		**156**		**181**		**223**	
	**G(+) *n = 8***	**G(−) *n = 8***	**G(+) *n = 8***	**G(−) *n = 8***	**G(+) *n = 7^**‡**^***	**G(−) *n = 8***	**G(+) *n = 8***	**G(−) *n = 8***
Catechin	907.9 (177.8)	812.4 (87.2)	572.4 (198.2)	592.3 (129.9)	166.3 (33.9)	210.3 (39.8)	82.9 (37.9)	104.7 (44.8)
Procyanidin B1	20.6 (8.2)^a^	10.8 (4.3)^b^	15.1 (1.2)	16.2 (3.2)	14.0 (1.2)	13.5 (3.8)	8.8 (1.6)	7.8 (2.2)
Epicatechin	354.0 (62.5)	342.7 (36.5)	300.4 (48.1)	347.2 (28.8)	158.5 (32.9)	181.4 (27.5)	99.8 (39.0)	108.8 (15.9)
Procyanidin B2	12.6 (4.3)	10.9 (1.3)	13.0 (2.4)	11.2 (4.3)	25.6 (3.7)	27.2 (10.2)	19.7 (3.9)	16.5 (3.6)
Epicatechin gallate	679.2 (31.1)	658.5 (48.0)	437.1 (93.5)	408.2 (101.0)	70.9 (7.2)	84.9 (18.9)	26.7 (13.2)	25.5 (4.9)
Polymeric Phenols	4227.9 (745.6)	4518.7 (236.1)	3958.9 (508.0)	4066.8 (152.4)	5122 (820.8)	5350.9 (725.6)	4964.3 (750.3)	5364.2 (495.3)
Total flavanols	6203	6355	5296	5441	5220	5868	5203	5629

*^1^Values are median (interquartile range, IQR) mg Kg^–1^ of seeds fresh weight; ^2^Phenological stages represented as days after pruning (DAP), where 146, veraison; 156, 30% maturation; 181, 70% maturation; 223, harvest; ^3^Grapevine received girdling application, G(+), or were non-girdled control, G(−). Differences between groups at each phenological stage were evaluated using the Mann Whitney *U*-test. Shaded groups with different letters are statistically significant (*p* < 0.05). *^‡^*One sample was lost during processing.*

### Multivariate Statistical Analysis

Metabolites from ^1^H NMR spectroscopy and HPLC-DAD data were visualized using principal components analysis (PCA) in order to identify which compounds had correlations and were most influenced by girdling. Metabolites in both tissues were strongly separated by maturation level, as was expected, while the effect of girdling was secondary. The best separation between girdled and non-girdled vine samples was observed with secondary metabolites (phenolics) determined in skins + pulp tissue, followed by phenolics in seeds (both determined using HPLC). Less separation was observed for primary and secondary metabolites determined in skins + pulp and seeds identified using ^1^H NMR spectroscopy.

## Discussion

### Impact of Girdling on Agronomical Data

**FIGURE 2:**
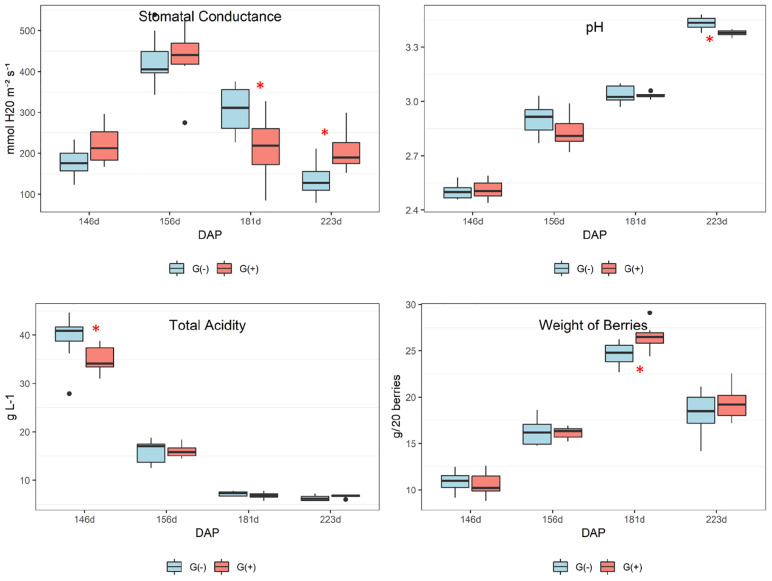
Agronomical parameters evaluated in leaves and grapes of ‘Cabernet Sauvignon’ at different phenological stages, from two treatments, girdled – G(+); and non-girdled – G(–) vines. Stomatal conductance was determined in vine leaves (*g*_s_); pH and total acidity were measured in grape berries. DAP = days after pruning, where: 146 corresponds to veraison; 156 to 30% maturation; 181 to 70% maturation; and 223 corresponds to harvest. Box plots show median, interquartile range (IQR), minimum/maximum, and strong outliers (>1.5 IQR). Differences between groups at each phenological stage were evaluated using the Mann Whitney *U*-test. Groups marked with (^∗^) are statistically significant (*p* < 0.05).

According to the agronomical parameters evaluated, stomatal conductance (g_s_) was significantly different between girdled and non-girdled vines. In both groups, the trend in g_s_ was an increase from veraison (146 dap, 0 dag) to 30% maturation (156 dap, 10 dag), followed by a reduction at 70% maturation (181 dap, 35 dag). At harvest, g_s_ was reduced further for (G-) at harvest and was significantly lower than G(+) (Figure 2). These findings demonstrate that girdled vines had higher rates of carbon dioxide entering or water vapor exiting through the stomata of the leaves at harvest, suggesting higher physiological activity. By using a leaf porometer, Roper and Williams (1989) showed that g_s_ was significantly lower for girdled table grape vines throughout most of the day, 4 weeks after girdling was applied. In the present study, we also showed reduced g_s_ for girdled vines at 70% berry maturation (181 dap), 35 days after girdling. However, at harvest (77 dag) results were contrary and girdled vines presented higher g_s_ as compared to non-girdled vines. These findings suggest higher stress and physiological activity for girdled vines close to harvest compared to non-girdled vines. Douthe et al. (2018) demonstrated that measurements of water and carbon fluxes at the whole-plant level under conditions mimicking the field presented results contrary to what occurs at the leaf scale. Stomatal conductance is helpful for studying interactions at the leaf scale as well as whole-plant-leaf dynamics (Buckley and Mott, 2013). As mentioned previously, the focus and objectives of this experiment was a metabolomics approach, which resulted in fewer eco-physiological measures being made, and future field studies should evaluate carbon fluxes of girdled and non-girdled vines using whole-plants.

Stomatal conductance behavior in most plants is regulated by hydraulic and chemical signaling, influencing guard cell physiology in response to water deficits and stress from other treatments, which is linked to abscisic acid and leaf water potential (Comstock, 2002). In one study, girdling reduced g_s_ in Canary Island pine (*Pinus canariensis*) at 140 dag, showing that the plant response to girdling may depend on the species (López et al., 2015). In this case, the authors suggested that the inhibitive effect of girdling on photosynthesis was primarily due to changes in the electron transport rate rather than changes in g_s_. Their two likely explanations for the negative feedback on photosynthesis was due either to an excess of starch grains leading to physical damage of thylakoids and the subsequent decrease of chlorophyll levels, or through the inhibition of photosynthetic genes regulated by carbohydrate content. The authors also found that girdling changed the concentration and ratio of photosynthetic pigments (chlorophyll concentrations progressively decreased in girdled plants), in addition to observing an acceleration of chlorosis in mature girdled leaves. In the current study, chlorosis was observed in both treatments and was more pronounced in leaves obtained from girdled vines at harvest (Figure 1, middle and bottom images).

The pH of grapes from G(−) was slightly higher than G(+) and values increased from veraison to harvest (Figure 2). This suggests differences in berry acidity, however, even though total acidity declined from veraison to harvest date, girdling did not influence total acidity in grapes. It is possible that other compounds may have influenced the pH of berries at harvest, such as organic acids, or that the balance of ions, titratable protons, and monovalent metal cations, such as potassium and sodium were altered (Boulton, 1980). Finally, berry weight increased from veraison (146 dap) to 181 dap (70% maturation), then decreased at harvest (no significant differences between girdled vs. non-girdled vines), with some shriveling observed. In the plots used for the current study, and in the majority of wineries throughout Napa Valley, grape harvesting occurs when sugars reach a minimum of 25°Brix. According to the boxplots of berry weights, shown in Figure 2, harvesting could be initiated 1−2 weeks earlier, or around 209−216 days after pruning, which could avoid volume losses associated with berry shriveling. Brar et al. (2008) showed that girdling at fruit set increased berry weight in table grapes, while Kennedy et al. (2000) showed that berries attained their maximum size approximately 30 days prior to harvest (24°Brix is considered commercially mature), with no evidence of berry contraction. The differences between our results and those from these authors are that the girdling in our study was applied at veraison and not at frut set, avoiding berry weight increases for wine grapes, as well as that in previous studies berries were harvested earlier before shriveling occurred. However, most Californian wineries harvest ‘Cabernet Sauvignon’ grapes later than this. Depending on harvest criteria, most commercial harvests may actually be taking place when fruit is overripe (>27°Brix), which would result in shriveled berries. Some authors showed that the volume loss in berries occurs due to declining phloem influx into the berries and cuticular berry transpiration (Coombe and McCarthy, 2000; Keller et al., 2006). It is possible that a similar effect occurred in the present study as a result of grapevine girdling, whereby phloem influx was reduced in order to balance water efflux; however, differences in berry weight between groups were not found in the current study.

### Impact of Girdling on Primary Metabolites in Skin/Pulp

Although all sugars (glucose, fructose, and sucrose) increased from veraison to harvest, there were no significant differences between groups at harvest in terms of sugars, organic acids, and carboxylic acids. Girdling was expected to increase the sugar content of berries, but no differences were found which may suggest the development of secondary phloem vessels that enabled sap movement from leaves to roots, as shown by others (Zhang et al., 2000). Zhang et al. (2000) showed that fascicular phloem is largely responsible for sugar transport, whereas the extra-fascicular phloem may function in signaling, defense, and the transport of other metabolites. Another study using a genomics approach showed leaf girdling induced leaf senescence and carbohydrate accumulation (Parrott et al., 2007), and girdling of a single leaf is observed to be sufficient for inhibiting photosynthesis and promoting starch accumulation, which in turn influences plant primary and secondary metabolism (Zhang et al., 2015). In addition, a study by Gallo et al. (2014) showed that agronomical practices such as girdling applied to table grapes affected primary metabolites, and increased the concentrations of sugar and the amino acid arginine.

**FIGURE 3:**
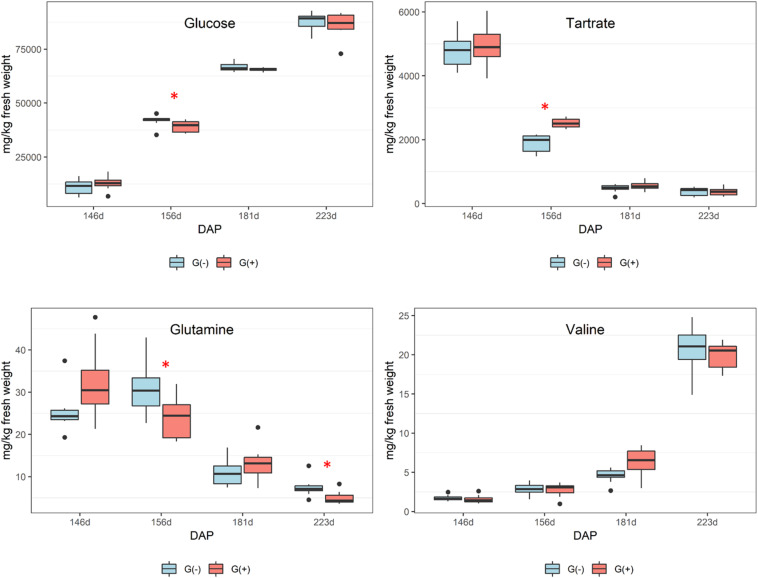
Metabolites determined by 1D ^1^H NMR spectroscopy in skin/pulp of ‘Cabernet Sauvignon’ grapes at different phenological stages, from two treatments, girdled – G(+); and non-girdled – G(–) vines. DAP = days after pruning, where: 146 corresponds to veraison; 156 to 30% maturation; 181 to 70% maturation; and 223 corresponds to harvest. Box plots show median, interquartile range (IQR), minimum/maximum, and strong outliers (>1.5 IQR). Differences between groups at each phenological stage were evaluated using the Mann Whitney *U*-test. Groups marked with (^∗^) are statistically significant (*p* < 0.05).

No differences in organic acids were found between treatments at harvest, and no clear trend was apparent. As expected, Figure 3 shows declining tartaric acid in skin/pulp from veraison to harvest, which likely occurred as a result of dilution, as shown in previous studies (Ollat et al., 2002). Significant differences in amino acids were only found for glutamine and threonine (Table 2). From veraison to harvest, glutamine was reduced in both groups, but girdling was shown to reduce glutamine concentrations at harvest. Some authors have shown that N composition of the phloem sap, particularly the glutamine content, may vary according to O_2_ diffusion and nitrogenase activity (Neo and Layzell, 1997). These authors showed evidence that the N content of phloem sap plays a role in the feedback regulation of nitrogenase activity, and that glutamine acts as a signal molecule regulating metabolism. Parrott et al. (2007) showed that genes associated with N metabolism, including glutamine synthetase, glutamate synthase, asparagine synthetase and several aminotransferase genes, were upregulated in girdled leaves. In tobacco leaves, glutamine was shown to be a precursor for the synthesis of proline via glutamate, and in the phloem played a major role as a key metabolite synthesized in response to water stress (Brugière et al., 1999).

In the current study, there was a trend toward higher amounts of proline, GABA, and other amino acids (results did not reach statistical significance) in non-girdled vines (Table 2). This could suggest the involvement of an alternate pathway for proline accumulation, which is one of the most important amino acids in grapes (Huang and Ough, 1991). Valine concentrations increased substantially from veraison to harvest, but no significant differences were found between treatments (Figure 3 and Table 2). Leucine, isoleucine, and tyrosine also substantially increased from veraison to harvest, contrary to the results shown by Lamikanra and Kassa (1999). This could be related to differences in demand for amino acids involved in protein synthesis that occur during grape ripening. Amino acids are known as important precursors for volatile and phenolic compounds (Gourieroux et al., 2016), and although high concentrations of amino acids can produce defective qualities in wines, they add complexity to wines at lower concentrations (Ardö, 2006).

### Impact of Girdling on Primary Metabolites in Seeds

**FIGURE 4:**
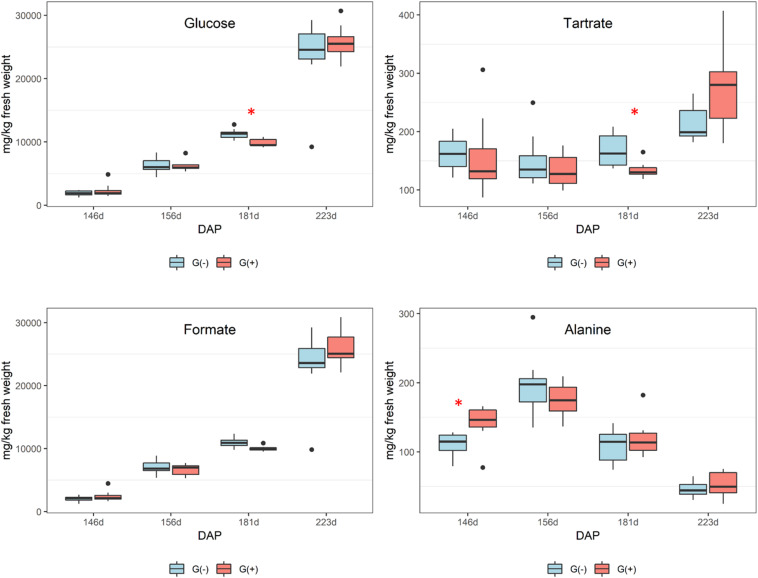
Metabolites determined by 1D ^1^H NMR spectroscopy in seeds of ‘Cabernet Sauvignon’ grapes at different phenological stages, from two treatments, girdled – G(+); and non-girdled – G(–) vines. DAP = days after pruning, where: 146 corresponds to veraison; 156 to 30% maturation; 181 to 70% maturation; and 223 corresponds to harvest. Box plots show median, interquartile range (IQR), minimum/maximum, and strong outliers (>1.5 IQR). Differences between groups at each phenological stage were evaluated using the Mann Whitney *U*-test. Groups marked with (^∗^) are statistically significant (*p* < 0.05).

Smaller variations were observed in seeds as compared to skin/pulp. Sugars accumulated from veraison in both treatments (Figure 4), but there were no differences between groups, except at 181 dap, in which glucose concentrations were observed to be higher in grapes grown on girdled vines. Others have shown that grape seeds are less influenced by abiotic factors than the skin and pulp tissues (Adams, 2006; Braidot et al., 2008; Carbonneau et al., 2015; Blancquaert et al., 2019). According to the ^1^H NMR spectroscopy data, the concentrations of organic acids and tartaric acid in grape seeds were stable from veraison to 181 dap, and then increased to harvest for both treatments (Figure 4), and tartaric acid concentrations were higher in G(−) vines at 181 dap as compared to G(+) vines (Table 3). Malic acid decreased from veraison to harvest, with no differences found between treatments. Bobeica et al. (2015) showed that the organic acids are less responsive to carbon limitation at harvest. Lamikanra and Kassa (1999) showed that amino acids content in skin, seed, pulp and grape berries presented different metabolite profiling from fruit set to harvest. A similar observation was made in the current study, in which alanine increased from veraison to 156 dap, but was reduced at harvest. Conversely, amino acids in skin/pulp trended toward increased proline, GABA, and other amino acids in girdled vines (Table 3).

### Impact of Girdling on Secondary Metabolites in Skin/Pulp

**FIGURE 5:**
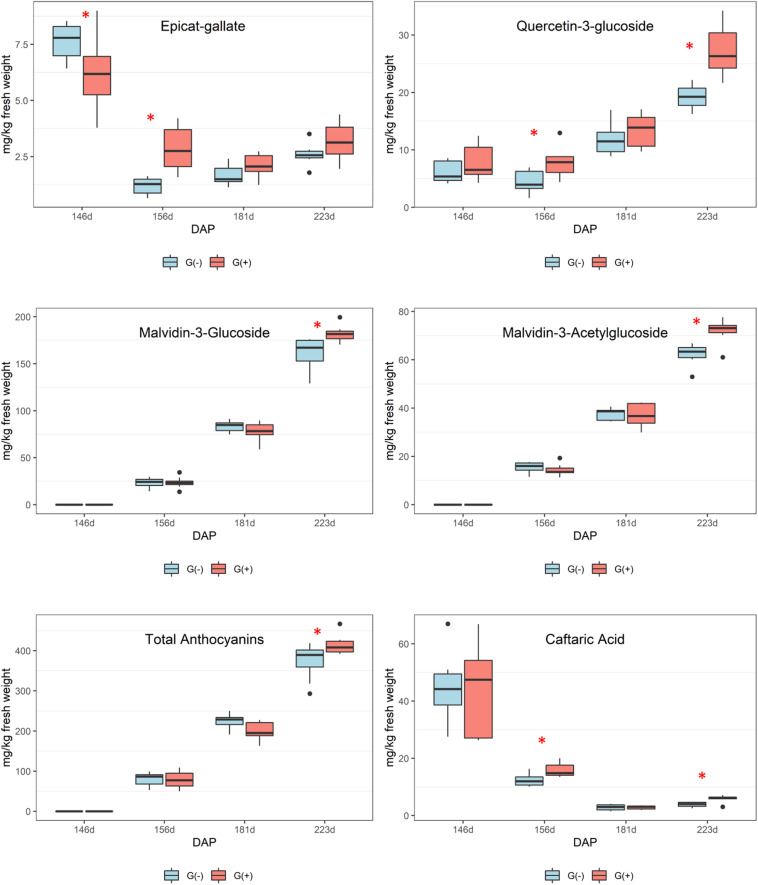
Metabolites determined by HPLC-DAD in skin/pulp of ‘Cabernet Sauvignon’ grapes at different phenological stages, from two treatments, girdled – G(+); and non-girdled – G(–) vines. DAP = days after pruning, where: 146 corresponds to veraison; 156 to 30% maturation; 181–70% maturation; and 223 corresponds to harvest. Box plots show median, interquartile range (IQR), minimum/maximum, and strong outliers (>1.5 IQR). Differences between groups at each phenological stage were evaluated using the Mann Whitney *U*-test. Groups marked with (^∗^) are statistically significant (*p* < 0.05).

Secondary metabolites were more influenced by girdling than primary metabolites as a greater number of significant results were found in skin/pulp grape extracts. Phenolics are complex compounds and are difficult to identify using 1D ^1^H NMR spectroscopy due to heterogeneous polymerization products and various hydrogen and carbon bonds (Fotakis et al., 2013; Lloyd et al., 2015). However, epicatechin concentrations, as determined by NMR spectroscopy, were higher in girdled compared to non-girdled vines. The HPLC data presented more significant findings as significant differences were observed for all phenolics at different phenological stages (Figure 5 and Table 4). Caftaric acid is a hydrocinnamic acid, specifically caffeic acid conjugated with tartaric acid, that was significantly higher in girdled vines at 30% maturation and at harvest. Some authors have reported a strong decrease in caftaric acid from veraison to harvest, and this metabolite is believed responsible for browning in raisins and wines (Singleton et al., 1985; Ali et al., 2010; Sun et al., 2017).

According to these results, it is important to highlight that girdled vines presented higher stomatal conductance at harvest, lower amounts of glutamine, a trend toward lower concentrations of other amino acids, and higher concentrations of the most important anthocyanins and flavonols in grapes, malvidin-3-glucoside and quercetin-3-glucoside, respectively. Many authors showed that lower fertilization and inputs of N for vines can be related to higher amounts of phenolic compounds in grapes and wines, and that abiotic factors can increase phenolics, with increased gene expression (Keller and Hrazdina, 1998; Bell and Henschke, 2005; Terrier et al., 2005; Keller et al., 2006; Van Leeuwen and Seguin, 2006; Braidot et al., 2008; Roullier-Gall et al., 2014; Sun et al., 2017). In the present study we can confirm a link between glutamine, a key signal regulating N accumulation, and the pathway involved in the biosynthesis of phenolic compounds, with higher concentrations of anthocyanins and flavonols in skin/pulp of ‘Cabernet Sauvignon’ grapes at harvest. Surprisingly, girdling did not affect sugar concentrations in mature ‘Cabernet Sauvignon’ grapes, potentially due to the development of extrafascicular vessels issued in the trunk zone; however, girdling did increase the concentration of phenolics. This increase was due to stress caused by girdling, because increased synthesis of phenolic compounds, including flavonoids and phenylpropanoid pathways, is a common plant response to stresses during grape ripening (Dixon and Paiva, 1995; Fortes et al., 2011).

### Impact of Girdling on Secondary Metabolites in Seeds

**FIGURE 6:**
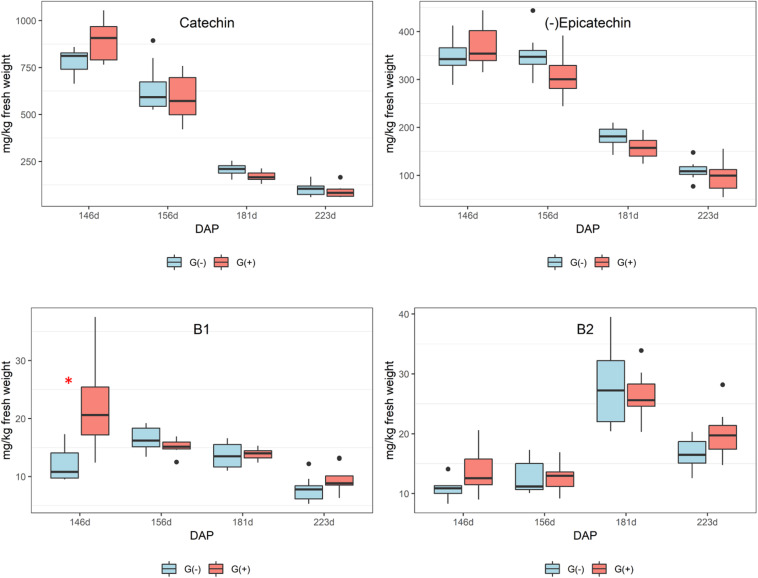
Phenolic compounds determined by HPLC-DAD in seeds of ‘Cabernet Sauvignon’ grapes from different phenological stages. DAP = days after pruning, where: 146 corresponds to veraison; 156 to 30% maturation; 181 to 70% maturation; and 223 corresponds to harvest. Box plots show median, interquartile range (IQR), minimum/maximum, and strong outliers (>1.5 IQR). Differences between groups at each phenological stage were evaluated using the Mann Whitney *U*-test. Groups marked with (^∗^) are statistically significant (*p* < 0.05).

In seeds, only epicatechin was identified and quantified by ^1^H NMR spectroscopy, and differences between girdled and non-girdled vines were not observed. For HPLC, flavan-3-ols were quantified in seed extracts of the treatments G(+) and G(−) from veraison to harvest, and similar to ^1^H NMR spectroscopy, significant differences were not observed for any metabolites (Table 5). There was a large decrease in catechin and epicatechin, while procyanidin B2 increased from veraison to 181 dap, followed by a decrease at harvest (Figure 6). Additional research is needed to identify this unknown compound, possibly using LC-MS or 2D NMR. Kennedy et al. (2000) also reported a dramatic decrease of 90% for flavan-3-ols during ripening and 60% for proanthocyanidins. The accumulation and alterations in skin and seed tannins are less understood, although it is clear that biosynthesis occurs mostly during the early stages of berry development, while the ripening phase is characterized by polymerization reactions and other alterations to existing tannin units (Keller et al., 2006). Different studies have shown that tannin concentrations differ according to grape variety (Ristic and Iland, 2005). Cadot et al. (2006) showed histologically that seed lignification is achieved at veraison, with proanthocyanidins localized in the epidermis while flavan-3-ol localization was linked with changes in cell walls of the outer integument.

In this study, girdling was applied at veraison and repeated after thirty days to increase the likelihood of the treatment being effective. We observed that it does not achieve additional gains in berry size and sugar level, but does fruit coloration (anthocyanins) and flavonols.

### Impact of Girdling on Metabolites by Multivariate Statistical Analysis

**FIGURE 7:**
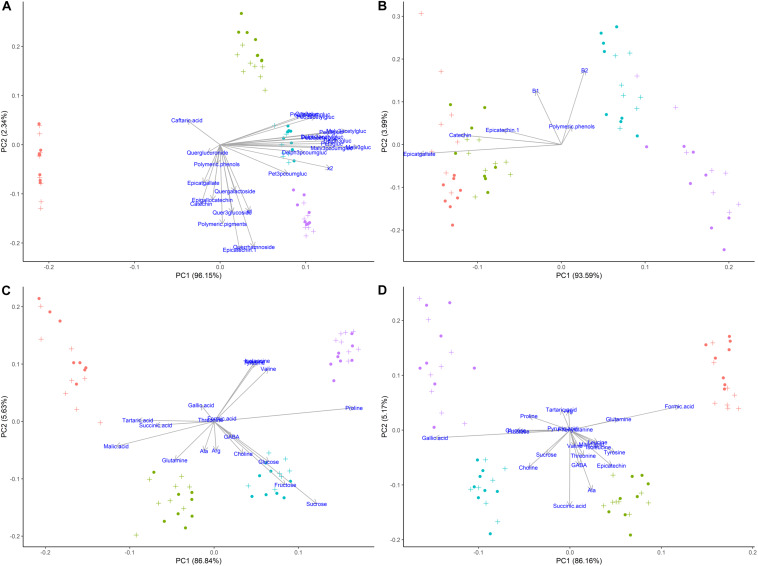
Principal component analyses of metabolites determined by HPLC-DAD and ^1^H NMR spectroscopy in different tissues of ‘Cabernet Sauvignon’ grapes at different phenological stages, from two treatments, girdled-G(+) and non-girdled vines- G(–). **(A)** HPLC-DAD of skin/pulp; **(B)** HPLC-DAD of seeds; **(C)** ^1^H NMR spectroscopy of skin/pulp; **(D)** ^1^H NMR spectroscopy of seeds. • = veraison (146 days after pruning, DAP); • = 30% maturation (156 DAP); • = 70% maturation (181 DAP); • = harvest (223 DAP); + = G(+); • = G(–).

According to the multivariate statistical analysis, PCA of skins + pulp data derived from the HPLC analysis showed that the first PC was responsible for 96.15% of total variability (Figure 7A). The most important factor responsible for sample discrimination was phenological stage, with samples at veraison (146 dap) separating strongly from samples collected at 30% maturation (156 dap), 70% maturation (181 dap), and harvest (223 dap). The main compounds driving separation were malvidin-3-glucoside and its derivatized forms, with higher concentrations on the right side of PC1. Figure 7B shows the PCA of metabolites in seeds derived from HPLC data. PC1 accounted for 93.59% of total variability, with grapes sampled at 146 and 156 dap separating from 181 and 223 dap. The metabolites driving this separation were epicatgallate, catechin and epicatechin, which were higher in grapes sampled from less mature vines (146 and 156 dap). Figure 7C shows the PCA of metabolites in skins + pulp tissue identified by ^1^H NMR spectroscopy. PC1 explained 86.84% of total variability, and once again strong separation was observed between samples from 146 dap and 156 dap and those from 181 dap and 223 dap. The main metabolites explaining sample variability for less mature grapes (146 and 156 dap) were malic acid, tartaric acid, and succinic acid, and glutamine, while proline, sucrose and valine were important for more mature grapes (181 and 223 dap). The PCA obtained from metabolites determined in seeds using ^1^H NMR spectroscopy was similar to skins + pulp data in that PC1 explained 86.16% of total variability and samples from 146 and 156 dap separated from grapes sampled at181 and 223 dap (Figure 7D). The main metabolites accounting for sample variability were formic acid and glutamine characterizing samples from veraison (146 dap), and gallic acid characterizing samples from harvest date (223 dap). Strongest separation was observed in secondary metabolites determined by HPLC in skins and pulps, followed by seeds. The main compounds driving separation were malvidin-3-glucoside and its derivatized forms. PCA of ^1^H NMR data shows that malic acid, tartaric acid, and succinic acid were driving separation early in the phenological development, and that proline and sucrose are of importance as grapes ripen. Hernández-Hierro et al. (2014) also showed that the most important factor discriminating polyphenols in grape samples harvested at different times and contents of soluble solids was the degree of ripeness.

**FIGURE 8:**
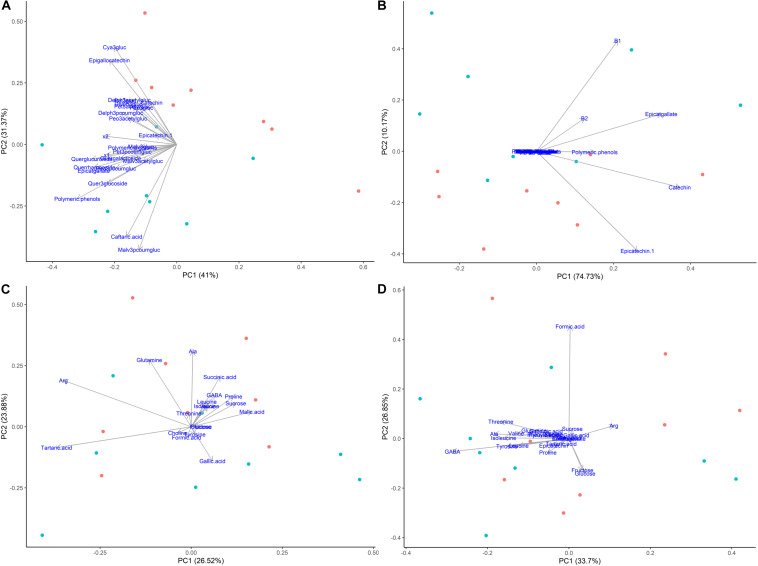
Principal component analyses of metabolites determined by HPLC-DAD and ^1^H NMR spectroscopy in different tissues of Cabernet Sauvignon grapes collected at harvest (223 days after pruning, DAP) from two treatments, girdled – G(+) and non-girdled vines - G(–). **(A)** HPLC-DAD of skin/pulp; **(B)** HPLC-DAD of seeds; **(C)** ^1^H NMR spectroscopy of skin/pulp; **(D)** ^1^H NMR spectroscopy of seeds. • = G(+); • = G(–).

Principal components analysis were also generated for each phenological stage, as ripening was the main driver for differences among samples when evaluating the whole sample set (Figure 7). Figure 8 shows the PCA of HPLC-DAD and ^1^H NMR spectroscopy generated data from grape tissue at harvest (223 dap). PCA of the other periods (146, 156 and 181 dap) did not show additional information. PCA of phenolic skins + pulp data (HPLC) showed treatment separation (girdled vs. non-girdled) in PC2, responsible for 31.37% of total variability (Figure 8A). PC1 showed only the sample variability. Treatment separation was due to cyanidin-3-glucoside and epigallocatechin, characterizing non-girdled skin/pulp at the top, while malvidin-p-coumaroylglucoside and caftaric acid characterized girdled samples, on the bottom of the graph. These results can be confirmed in Table 4. Figure 8B shows the PCA of phenolic metabolites in the seeds (HPLC), but clear separation due to treatment were not obtained, similar to skin + pulp and seed metabolites determined by ^1^H NMR spectroscopy (Figures 8C,D). Results indicate that girdling impacted mainly the secondary metabolites in the skin + pulp.

The metabolomics approach carried out in this study provided further information regarding vine development and skin, flesh/pulp and seed metabolite accumulation in Cabernet Sauvignon grapes from veraison to harvest. Additional research should be carried out (i) to evaluate the use of girdling at fruit set with the aim of achieving greater balance in the composition of sugars, a higher anthocyanins and tannins contents, and to harvest an earlier crop. New studies could also evaluate (ii) the effect of girdling on volatile compounds using GC-MS. Further, studies are needed (iii) to determine the optimal date for harvest, in terms of berry weight reduction versus wine characteristics. For this, focus should be placed on larger sample sizes at the period of 70% maturation to harvest. Also, (iv) several unidentified compounds in skins/pulp and seeds could be elucidated with the use of 2D NMR (^1^H-^1^H and/or ^1^H-^13^C). Finally (v), future work could utilize genomic approaches to identify genes involved in primary and secondary pathways in grape berries after girdling, between veraison and harvest. Future research should also investigate the impact of girdling over multiple seasons and cultivars, as a metabolic effect has been shown.

## Conclusion

A metabolomics approach was used to evaluate the effect of grapevine girdling on vine development, and metabolite accumulation in the skins/pulp and seeds of ‘Cabernet Sauvignon’ grapes, from veraison to harvest. Girdling at veraison increased stomatal conductance in vines at harvest, decreased glutamine, and increased anthocyanin and flavonol concentrations in skin/pulp tissues of grape berries, while primary metabolites such as sugars, organic acids, and other amino acids in skin/pulp and seeds were not dramatically affected. We hypothesize that this is due to extrafascicular phloem vessels transporting metabolites from leaves to the roots in vines. Girdling is a simple technique that could be used commercially for vine management to improve berry enological potential, particularly in terms of promoting the development of anthocyanins and flavonols in ‘Cabernet Sauvignon’ grapes.

## Data Availability Statement

All datasets generated for this study are included in the article/supplementary material.

## Author Contributions

SK and AO provided the experiment plan. GP, RG, DT, RB, CM-P, JE, and AO carried out agronomical and physiological measurements, berry samplings, and harvest. GP performed the extractions on grape berries. CS provided ^1^H NMR spectroscopy sample preparation support. GP and EP analyzed the 1D ^1^H NMR spectroscopy data. GP and CM-P performed the HPLC analysis. EP performed the statistical analyses. GP, AO, and EP interpreted the results. GP wrote the manuscript. AO and EP edited the manuscript. All authors read and approved the final manuscript.

## Conflict of Interest

GP was employed by company Brazilian Agricultural Research Corporation-Embrapa Grape & Wine. The authors declare that the research was conducted in the absence of any commercial or financial relationships that could be construed as a potential conflict of interest.
